# Systematic Review and Meta-analysis of Foodborne Tick-Borne Encephalitis, Europe, 1980–2021 

**DOI:** 10.3201/eid2810.220498

**Published:** 2022-10

**Authors:** Meital Elbaz, Avi Gadoth, Daniel Shepshelovich, David Shasha, Nir Rudoler, Yael Paran

**Affiliations:** Tel Aviv Sourasky Medical Center, Tel Aviv, Israel (M. Elbaz, A. Godath, D. Shepshelovich, D. Sasha, Y. Paran);; Tel-Aviv University, Tel Aviv (A. Godath, D. Shepshelovich, D. Sasha, Y. Paran);; Hebrew University, Jerusalem, Israel (N. Rudoler); Ministry of Health, Tel Aviv (N. Rudoler)

**Keywords:** Tick-borne encephalitis, meningitis/encephalitis, vector-borne infections, food safety, zoonoses, viruses, foodborne diseases, alimentary infection, unpasteurized milk, attack rate, Europe, Israel

## Abstract

Tick-borne encephalitis (TBE) is a viral infection of the central nervous system that occurs in many parts of Europe and Asia. Humans mainly acquire TBE through tick bites, but TBE occasionally is contracted through consuming unpasteurized milk products from viremic livestock. We describe cases of TBE acquired through alimentary transmission in Europe during the past 4 decades. We conducted a systematic review and meta-analysis of 410 foodborne TBE cases, mostly from a region in central and eastern Europe. Most cases were reported during the warmer months (April–August) and were associated with ingesting unpasteurized dairy products from goats. The median incubation period was short, 3.5 days, and neuroinvasive disease was common (38.9%). The clinical attack rate was 14% (95% CI 12%–16%), and we noted major heterogeneity. Vaccination programs and public awareness campaigns could reduce the number of persons affected by this potentially severe disease.

## Abstract 2

Most cases were associated with ingesting unpasteurized dairy products from goats; the clinical attack rate was 14%.

Tick-borne encephalitis (TBE) is a viral infection of the central nervous system (CNS) caused by tick-borne encephalitis virus (TBEV). TBE occurs mainly in eastern, central, and northern Europe and in northern China, Mongolia, and Russia (*1*). Although vaccination can effectively prevent TBE, >3,000 cases were reported in Europe in 2019, and case-fatality was 0.7% (*2*). However, many mild and subclinical infections probably remain undiagnosed.

Humans acquire TBE mainly via tick bites, but TBEV can occasionally be transmitted through consumption of unpasteurized milk products from viremic livestock. The largest known outbreak of foodborne TBE (FB-TBE) occurred in 1954 in what was then Czechoslovakia, when TBE developed in >600 persons infected via TBEV-contaminated milk from cows and goats (*3*). During that period, the disease was termed biphasic milk fever. During the past 4 decades, repeated smaller outbreaks have been reported in association with TBEV transmission via contaminated milk in various countries in Europe and in Russia (*3*–*10*).

Despite the role of food as a transmission route, FB-TBE has not been systematically described until recently. Two recent published reviews summarized published reports (*11*,*12*), but those studies did not include meta-analysis of published data. We systematically describe cases of alimentary TBEV transmission in Europe during the past 4 decades, estimate the attack rate of FB-TBE, and describe the epidemiologic and clinical characteristics of FB-TBE.

## Methods

We conducted a systematic review and meta-analysis according to guidelines of Preferred Reporting Items for Systematic Reviews and Meta-Analyses (PRISMA, http://www.prisma-statement.org) (*13*). We searched articles published during January 1, 1980–June 1, 2021, on PubMed (https://pubmed.ncbi.nlm.nih.gov) and Embase (https://www.embase.com) databases using the following key terms: (“tick-borne encephalitis” OR “TBE”) AND (“food” OR “alimentary” OR “milk” OR “cheese” OR “dairy”). We excluded duplicate publications and articles without available abstracts. We screened all publications and selected those that met our eligibility criteria. We did not restrict inclusion by study type or minimum number of patients.

We only included original studies on human data for confirmed and probable cases of FB-TBE that were published in English. We reviewed and extracted data from articles meeting eligibility criteria. We collected data on the number of persons exposed to contaminated products, number of confirmed FB-TBE cases, laboratory testing, source of infection, geographic location, year and season of outbreak, incubation period, vaccination status, and clinical signs and symptoms of invasive CNS disease, when reported.

### Definitions

We defined a confirmed FB-TBE case as a positive laboratory test supporting TBEV infection in a person with or without symptoms of infection (Appendix Table) who also had a possible link to consumption of raw milk or cheese and did not recall having a tick bite. We defined a probable FB-TBE case as a person with symptoms compatible with TBE that was not tested for the virus but who was exposed to raw milk or cheese and did not recall a tick bite; probable cases were included only when a cluster of >2 exposed persons and virologic confirmation for TBE were reported.

We defined confirmed invasive CNS disease (meningitis, meningoencephalitis, or myelitis) when CNS neurologic symptoms (e.g., headache, vomiting, ataxia, altered consciousness, confusion, dysphasia, or hemiparesis) were reported and laboratory testing confirmed TBEV infection, including TBEV-positive cerebrospinal fluid (CSF) serology or CSF pleocytosis and other laboratory testing supporting TBEV infection. We defined probable invasive CNS disease as a combination of CNS neurologic symptoms and laboratory-confirmed TBEV infection in a patient who did not undergo lumbar puncture and CSF analysis.

### Outcomes

 For studies recording the number of persons exposed to TBEV-contaminated dairy products, we calculated the FB-TBE clinical attack rate by dividing the number of symptomatic patients with confirmed and probable TBE by the number of persons exposed to the same dairy products. We also describe epidemiologic features, including country, season, and source of infection, and clinical characteristic including incubation period, vaccination status, and whether patients had biphasic disease or CNS involvement.

### Statistical Methods

We pooled attack rates by using meta-analysis for untransformed proportion in a DerSimonian and Laird fixed-effects model. We assessed the level of heterogeneity (*I^2^*) by visually examining the forest plot for nonoverlapping CIs and using χ^2^ test. We considered p<0.05 statistically significant and *I^2^* >50% substantially heterogenic.

## Results

**Figure 1:**
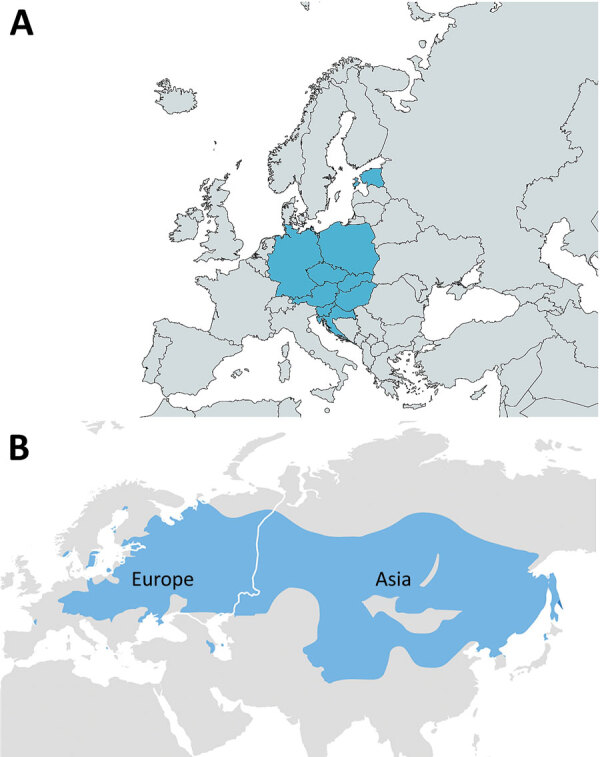
Geographic distribution of reported foodborne tick-borne encephalitis (FB-TBE) cases (blue shading), Europe, 1980–2021. A) The FB-TBE triangle in Europe. Russia had 5 cases in 1991 (not shown). Map created by using MapChart (https://mapchart.net). B) The tick-borne encephalitis belt, spanning from western Europe, across Russia, China, and Mongolia to Japan. Map from the Centers for Disease Control and Prevention (https://www.cdc.gov/tick-borne-encephalitis/geographic-distribution/index.html).

Our search retrieved 61 articles, including 25 reporting the same outbreaks. Of the remaining 36 articles, 10 reported nonhuman outbreaks, 4 were in languages other than English or had no abstract available, and 3 overlapped with other studies. Ultimately, we included 19 studies meeting eligibility criteria, describing 410 patients across Europe: 384 (94%) with confirmed FB-TBE and 26 (6%) with probable FB-TBE. Countries reporting FB-TBE cases during 1980–2021 included Slovakia (*5*,*14*,*15*), the Czech Republic (*3*,*16*), Poland (*17*,*18*), Hungary (*10*,*19*), Estonia (*8*,*20*), Germany (*21*,*22*), Croatia (*23*,*24*), Austria (*9*,*25*), Russia (*6*), and Slovenia (*26*) (Table 1; Figure 1).

**Table 1:** Foodborne and nonfoodborne TBE cases, Europe, 1980–2021*

Country, y (reference)	Total FB-TBE cases	No. FB-TBE/TBE cases (%)†
Slovakia (*5*,*14*,*15*)	177	
1993		7/NA
2012		15/32 (46.88)
2013		5/157 (3.18)
2014		11/115 (9.57)
2015		14/80 (17.50)
2016		65/169 (38.46)
2009–2016		60 additional cases not included in mentioned outbreaks‡
Czech Republic (*3*,*16*)	65	
1994		1/617 (0.16)
1997		2/415 (0.48)
1998		1/422 (0.24)
1999		28/489 (5.73)
2002		5/647 (0.77)
2003		6/606 (0.99)
2004		2/507 (0.39)
2005		8/643 (1.24)
2007		8/546 (1.47)
2008		4/631 (0.63)
Poland (*17*,*18*)	52	
1995		48/NA
2017		4/196 (2.04)
Hungary (*10*,*19*)	42	
2007		31/69 (44.93)
2011		11/43 (25.58)
Estonia (*8*,*20*)	28	
2005		27/164 (16.46)
2019		1/82 (1.22)
Germany (*21*,*22*)	16	
2016		2/348 (0.57)
2017		14/485 (2.89)
Croatia (*23*,*24*)	14	
2015		9/26 (34.62)
2019		5/13 (38.46)
Austria (*9*,*25*)	8	
1989		2/NA
2008		6/86 (6.98)
Russia (*6*)	5	
1991		5/NA
Slovenia (*26*)	3	
2012		3/164 (1.83)

*FB-TBE, foodborne tick-borne encephalitis; NA not available; TBE, tick-borne encephalitis. †Number of TBE cases are from European Centre for Disease Prevention and Control annual report (*2*) and other reports (*27*,*28*). ‡From (*14*).

Of 273 patients with data regarding the season of infection, 243 (89%) were infected during April–August and 30 (11%) during September–November. Patient age distribution was wide, 1–85 years. Of the 120 FB-TBE patients for whom vaccination status was recorded, only 1 was vaccinated (*21*). The 1 exposed and vaccinated person had their last TBEV vaccination booster >15 years before infection; thus, the booster was >10 years overdue.

Among 232 (66%) patients, epidemiologic investigation revealed consumption of raw goat milk or cheese; consumption of raw sheep milk or cheese was reported in 88 (25%) cases, consumption of unpasteurized cow milk in 23 (7%) cases, and consumption of a mixture of unpasteurized dairy products in 7 cases (2%). For 124/138 (90%) patients for whom incubation period was reported, incubation was <2 weeks. Among 14 patients who reported the exact infection timeline, the median incubation period was 3.5 days (IQR 2–14 days).

Biphasic disease was reported in 49/64 (77%) patients for whom the disease course was described. Common symptoms of the first phase included nonspecific influenza-like symptoms, fever, vomiting, loose stools, headache, bilateral orbital pain, vertigo, sore throat, chills, bone pain, myalgia, and malaise.

Proven neuroinvasive disease was documented in 53/136 (39%) patients in the 13 studies that specifically reported on CNS disease (Table 2). Probable CNS invasive disease was reported in 24 additional cases, making the rate of probable and proven neuroinvasive disease 56% (77/136 patients). Among 23 patients for whom CNS syndrome was described, 13 (57%) patients had diagnosed meningoencephalitis, 9 (39%) had meningitis, and 1 (4%) had meningoencephalomyelitis. Diagnosis of proven CNS disease was made by positive CSF serology in 45 (87%) patients, and CSF pleocytosis and positive serum serology in 7 (13%) patients (Table 2).

**Table 2:** Neuroinvasive disease reported in cases of foodborne tick-borne encephalitis, Europe, 1980–2021*

Country (reference)	No. confirmed cases	No. CNS invasive disease	CNS invasive disease type	Blood serology	CSF serology
Austria (*9*)	6	4	4 ME cases	Positive IgG and IgM	Borderline IgM, positive IgG; borderline IgM, borderline IgG; positive IgM, positive IgG; positive IgM, borderline IgG
Croatia (*23*)	7	6 proven, 7 symptomatic	5 meningitis cases, 1 ME case; 1 case with fever and headache but LP not performed	Positive IgG and IgM	6 patients had CSF pleocytosis but negative IgG and IgM
Czech Republic (*16*)	1	1	ME, myelitis	Positive IgM	CSF pleocytosis
Estonia (*20*)	1	1	ME	Positive IgM and IgG	positive serology
Germany (*22*)	2	2	ME in both cases	Positive IgG and IgM	Positive IgG and IgM
Hungary (*10*)	25	2 confirmed; 25 with neurologic symptoms but LP only performed on 3		Positive IgG and IgM	Positive IgG in 2 of 3 CSF samples
Hungary (*19*)	7	4	4 confirmed ME cases	In all 7 confirmed cases, positive IgM in blood or CSF	
Poland (*18*)	35	15		Positive IgM and IgG	Positive serology for 15 patients with neuroinfection
Poland (*29*)	4	4	4 meningitis cases	2 had elevated IgG and IgM. 1 had only elevated IgM. The fourth wasn't examined	All 4 had elevated IgG and IgM
Slovakia (*15*)	2	1		Positive IgM	Positive IgM
	43	12		Positive IgM and IgG	12 patients with IgM and IgG in CSF
Slovenia (*26*)	3	1	2 cases with symptoms of ME but LP only performed on 1	Positive IgG and IgM	CSF pleocytosis

*CNS, central nervous system; CSF, cerebrospinal fluid; LP, lumbar puncture; ME, meningoencephalitis.

**Figure 2:**
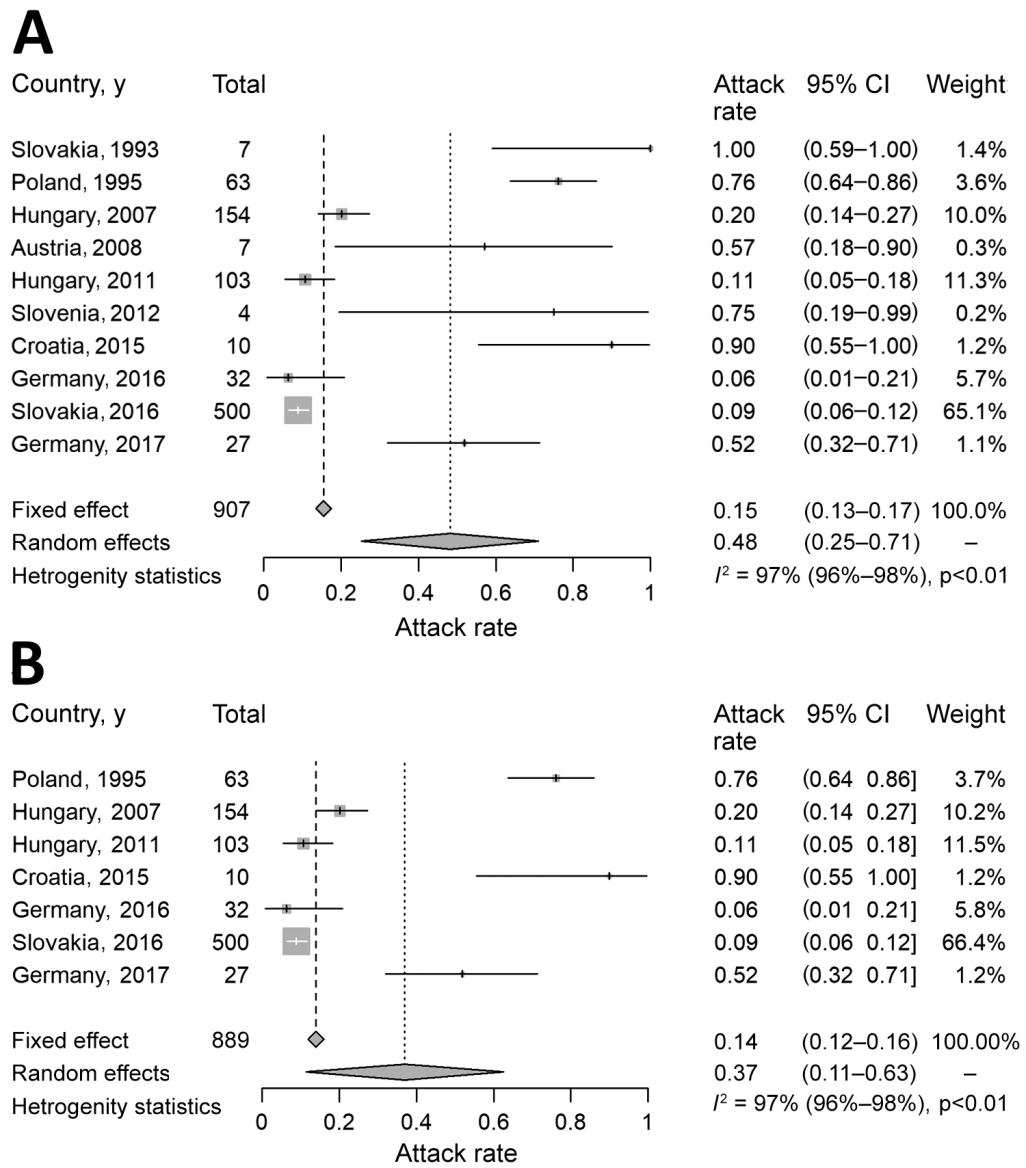
Analysis of clinical attack rate of foodborne tick-borne encephalitis, Europe, 1980–2021. A) Attack rate calculated for 10 outbreaks in which the number of exposed persons was reported. B) Attack rate calculated only for 7 outbreaks with >10 reported persons affected. *I^2^*, level of heterogeneity.

We calculated attack rates for 10 outbreaks in which the number of exposed persons was reported (Table 3), representing a total of 907 exposed persons. We found a wide range of attack rates, from 6% in Germany in 2016 to 100% in Slovakia in 1993. The pooled attack rate was 15% (95% CI 13%–17%). Heterogeneity was significant (*I*^2^ = 97.4%, 95% CI 96.5%–98.1%; p<0.01) but yielded inconsistent results, making *I*^2^ an unreliable attack rate estimator (Table 3; Figure 2).

**Table 3:** Attack rates for foodborne tick-borne encephalitis, Europe, 1980–2021

Country (reference)	Year	No. persons exposed	Clinical attack rate, %	Source of dairy products
Slovakia (*5*)	1993	7	100	Goat
Poland (*18*)	1995	63	76.2	Goat
Hungary (*10*)	2007	154	20.1	Goat
Austria (*9*)	2008	7	57.1	Goat and cow
Hungary (*19*)	2011	103	10.7	Cow
Slovenia (*26*)	2012	4	75	Goat
Croatia (*23*)	2015	10	90	Goat
Germany (*22*)	2016	32	6.3	Goat
Slovakia (*15*)	2016	500	8.8	Sheep
Germany (*21*)	2017	27	51.9	Goat

We applied an additional meta-analysis that included outbreaks with >10 reported cases, representing 7 outbreaks and a total of 889 exposed persons (Table 3). We still found a wide range in attack rates, from 6% in Germany in 2016 (*22*) to 90% in Croatia in 2015 (*23*). The pooled attack rate was 14% (95% CI 12%−16%) (Figure 2). Heterogeneity was significant (*I*^2^ = 97.5%, 95% CI 96.3%–98.3%; p<0.01) but again yielded inconsistent results and demonstrated *I^2^* is an unreliable attack rate estimator.

**Figure 3:**
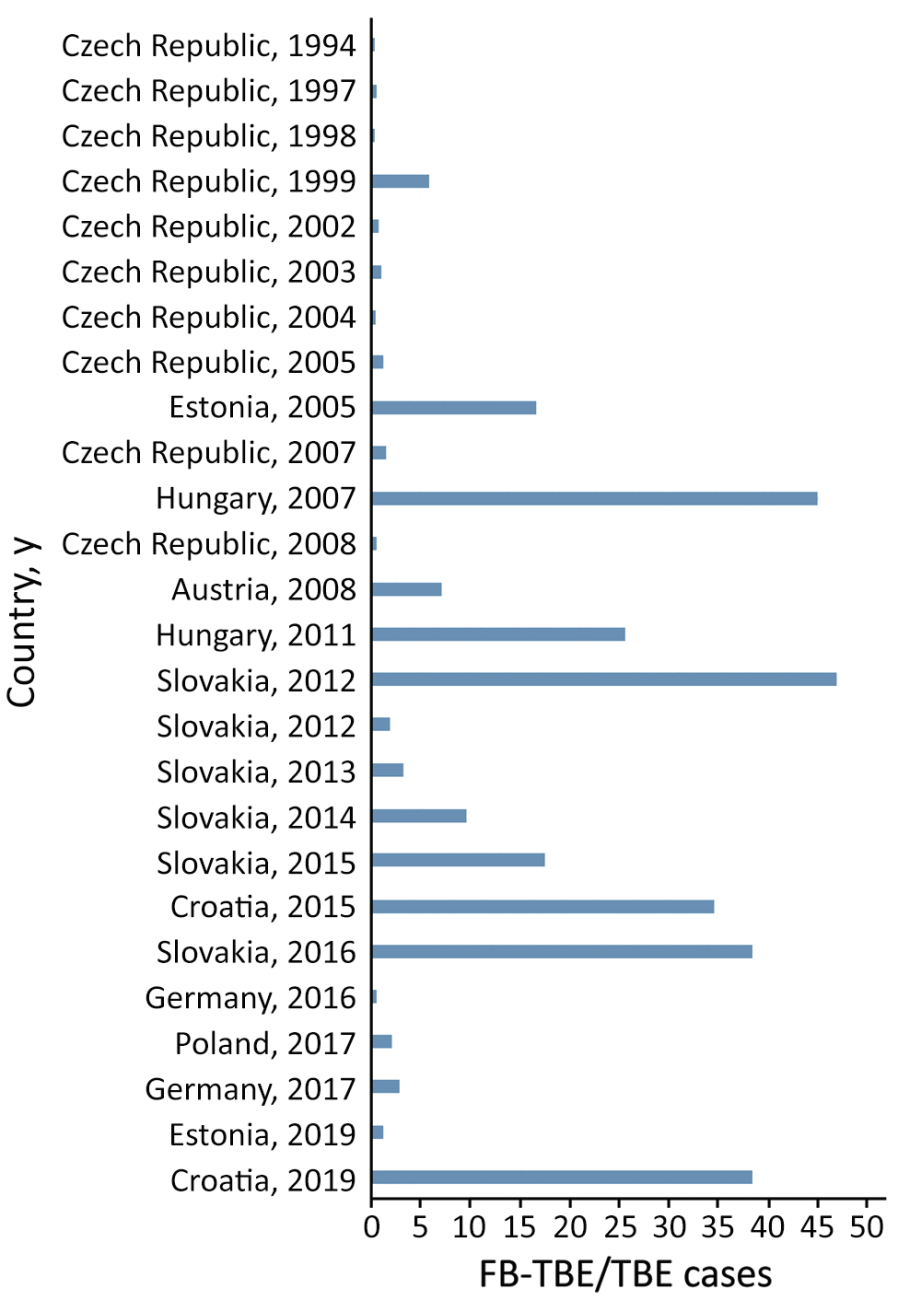
Rates of FB-TBE per country and year from a systematic review and meta-analysis, Europe, 1980–2021. Rates indicate FB-TBE per all TBE cases reported in the country, per year. Data are based on those reported in Table 1. FB-TBE, foodborne tick-borne encephalitis; TBE, tick-borne encephalitis.

For 26 outbreaks, we calculated the rate of FB-TBE out of all reported TBE cases in the country each year (Table 1; Figure 3). We calculated the median rate of FB-TBE only for outbreaks occurring after 2012, when TBE became a notifiable disease in the European Union (*30*). The median rate of FB-TBE per TBE cases was 6% (IQR 2%–36%).

## Discussion

We report 410 cases of FB-TBE, most from a region in central and eastern Europe stretching from Croatia in the south to Poland and Germany in the north and an anecdotal report of 5 cases in Russia (*6*). This region represents an FB-TBE triangle (Figure 1, panel A). Most cases were reported during the warmer months of April–August and were associated with ingestion of unpasteurized dairy products from goats. None of the infected patients were vaccinated, except 1 patient who had their last TBE vaccination booster >15 years before infection. FB-TBE incubation period was short (median 3.5 days), and invasive CNS disease was common. The clinical attack rate was 14% (95% CI 12%−16%) for outbreaks with >10 reported cases and heterogeneity was significant (*I*^2^ = 97.5%).

Although TBE is a mandatory reportable disease in Europe (*30*,*31*) and cases of TBE are distributed along the southern part of the nontropical Eurasian Forest belt (Figure 1, panel B), we noted that nearly all FB-TBE cases occurred in a region we termed the FB-TBE triangle (Figure 1, panel A). This phenomenon might be explained by different habits of consumption of unpasteurized dairy products in different regions, but data on the frequency of unpasteurized dairy consumption in various parts of Europe is lacking. Of note, geographic distribution of brucellosis, a zoonosis caused by ingestion of unpasteurized milk, is not concordant with distribution of FB-TBE. One explanation for this discrepancy is that brucellosis is transmitted not only by raw dairy consumption but also by consuming undercooked meat and by contact with body fluids from farm animals. Moreover, brucellosis is a preventable disease by national eradication programs and vaccination of cattle in areas with high prevalence. For example, the Czech Republic has high FB-TBE rates but had no reported brucellosis cases during 2013–2017 (0.00 cases/100,000 population), according to a European Centre for Disease Prevention and Control annual report (*32*), likely resulting from a brucellosis eradication program among livestock, which was successfully completed in 1964.

TBEV has 3 subtypes: European (TBEV-Eu), Siberian (TBEV-Sib), and Far Eastern (TBEV-FE). The vector of TBEV-Eu is *Ixodes ricinus* ticks; *I. persulcatus* ticks are the vectors for the other 2 subtypes (*33*). *I. ricinus* ticks are seen in most of Europe, and their distribution extends to Turkey, northern Iran, and the Caucasus in the southeast (*34*). *I. persulcatus* ticks are found in the belt extending from eastern Europe to China and Japan. Both tick species circulate in a restricted area in northeastern Europe; northern areas of the Republic of Karelia in Russia; St. Petersburg, Russia; eastern Estonia; and eastern Latvia (*35*,*36*). Consequently, all 3 TBEV subtypes have been recorded in these regions. In all the countries of the FB-TBE triangle, the TBEV-Eu subtype dominates, except in Estonia. Possible explanations could be underreporting of FB-TBE in the countries where the TBEV-Sib and TBEV-FE subtypes dominate due to unawareness of foodborne transmission or different habits of raw dairy consumption. Another explanation could be different capabilities for the vector or the virus to infect livestock or to survive in dairy products.

Recently, an outbreak of encephalitis and meningoencephalitis occurred in the Ain department of eastern France, where TBEV had not previously been detected (*37*). Epidemiologic investigation revealed that all but 1 of 43 TBE patients with encephalitis, meningoencephalitis, or influenza-like symptoms had consumed unpasteurized goat cheese from a single local producer. The researchers confirmed the alimentary origin of the TBE outbreak, and phylogenetic analyses found that the strain involved, TBEV_Ain_2020, belongs to the TBEV-Eu subtype (TBEV-Eu3) and is most closely related to TBEV strains recently isolated in bordering countries and eastern Europe. This finding emphasizes the role of foodborne transmission in TBE, even in areas where TBEV has never been detected. In addition, this finding is compatible with our observation of an association between the TBEV-Eu subtype and foodborne transmission.

We found that FB-TBE attack rates ranged widely. Possible explanations for the wide range could be underdiagnosis, underreporting, variations due to the low number of patients involved in some of the reports, and incomplete epidemiologic investigations. An alternative explanation might be the variability in the viral load in the infected dairy products because the exact TBEV dose required for human infection via the oral route is unknown and might be different from the viral load required for clinical infection through tick bites. Outbreaks with low attack rates might not have had high portions of milk or cheese that contained enough TBEV to cause human infection. In an analysis of cheese from the manufacturing and storage facilities of a dairy farm responsible for an outbreak, quantitative reverse transcription PCR and isolation results implied that the distribution of TBEV loads in infected goat cheese was heterogenous (*22*), which likely contributes to the variability in attack rate we observed.

Although alimentary transmission of TBE is uncommon, this transmission mode has the potential to cause outbreaks affecting many persons, making FB-TBE a major public health concern. Foodborne transmission could easily be eliminated through education campaigns that encourage persons to consume only pasteurized dairy products and through vaccination. Vaccination seems to be effective in preventing FB-TBE, not only disease caused by tick bites. In our cohort, among 120 FB-TBE patients for whom vaccination status was recorded, only 1 was vaccinated but did not receive an appropriate booster. Other observations regarding the effectiveness of the vaccine against alimentary transmission were made during a 2017 FB-TBE outbreak that included 27 exposed persons (*21*). Among 20 persons for whom medical information was available, 13 were infected and reported symptomatic disease. Among 6 exposed persons who were vaccinated, only 1 person developed disease, but that person was vaccinated >15 years prior to exposure. In contrast, among 14 unvaccinated exposed persons, 12 had TBE develop (*21*). Findings from that outbreak suggest that vaccination also protects against alimentary TBEV transmission.

Most reported FB-TBE cases were documented in months that parallel tick season in Europe, even though transmission was through ingestion of contaminated dairy products. This finding probably implies that the infected livestock are most viremic during the peak of tick season.

We found the median incubation time for FB-TBE was shorter (median 3.5 days) than that for non–FB-TBE, which was reported to be 8 days (range 4–28) in 1 study (*1*). Another study reported a much longer incubation period, median 22 days, in 687 patients in Poland (*29*). We suggest that symptoms compatible with TBE in the context of recent exposure to raw dairy products should raise suspicion of FB-TBE, especially when symptoms develop in >1 patient exposed to the same source. This finding can assist clinicians and help guide epidemiologic investigation.

Although the transmission mode is different and the incubation period is shorter, FB-TBE has clinical manifestations similar to those for disease transmitted by ticks, and most symptomatic patients experience biphasic disease, as described (*1*,*38*). Among patients with CNS involvement, most had meningitis or meningoencephalitis, and myelitis was a rare manifestation, comparable with previous reports of TBE (*38*).

Typically, TBE is biphasic and 70% of patients experience neuroinvasive disease (*1*,*39*). We found lower rates of invasive disease in FB-TBE; only 39% of patients had neuroinvasive disease. Actual rates of neuroinvasive disease in TBE are challenging to assess because patients with mild symptoms and no CNS-specific symptoms are less likely to seek medical care; even for patients who do seek care, many will have diagnoses of nonspecific viral syndrome. FB-TBE outbreaks can help determine the actual rate of neuroinvasive disease because epidemiologic investigation of patients exposed to a common source can actively locate patients with only mild and nonspecific symptoms.

The first limitation of this study is that, although TBE is a reportable disease in many countries in Europe, many cases are not reported or are misdiagnosed by clinicians because most infected persons who experience clinical disease have only mild nonspecific symptoms, which could lead to underestimation of the true number of TBE cases. Moreover, ≈30%–50% of patients with diagnosed TBE do not recall a tick bite, but probably few are asked about consumption of unpasteurized dairy products, which could lead to underestimation of FB-TBE. Even in cases where epidemiologic investigations were conducted, many exposed persons might remain unidentified and untested for the reasons we mentioned, making the true attack rate higher than calculated here. Assessment of the attack rate was also limited by high variability between studies. Finally, we included only published articles and not reports from ProMED (https://promedmail.org) or other sources, and we almost certainly missed some FB-TBE cases.

In conclusion, FB-TBE in Europe is reported mostly in a well-defined geographic region during tick season, with a few reports from Russia and recently in France. We found a variable FB-TBE attack rate, which might be the result of many factors, including variability in the viral load in the infected dairy products, compatible with a previous report (*22*). Clinical features of FB-TBE are similar to those reported for TBE acquired through tick bites, and CNS-specific symptoms develop in nearly 40% of infected persons. Vaccination seems to be effective in preventing FB-TBE. Our findings could help raise awareness of FB-TBE among epidemiologists, clinicians, public health officials, and the public in endemic areas. Vaccination programs and public awareness campaigns could greatly reduce the number of patients affected by this potentially severe CNS infection.

Appendix Additional information on foodborne tick-borne encephalitis, Europe, 1980–2021.

## References

[1] Lindquist L, Vapalahti O. Tick-borne encephalitis. Lancet. 2008;371:1861–71. 10.1016/S0140-6736(08)60800-4 18514730

[2] European Centre for Disease Prevention and Control. Tick borne encephalitis. In: Annual epidemiological report for 2019. Stockholm: The Centre; 2021.

[3] Kríz B, Benes C, Daniel M. Alimentary transmission of tick-borne encephalitis in the Czech Republic (1997-2008). Epidemiol Mikrobiol Imunol. 2009;58:98–103. 19526924

[4] Gresíková M, Sekeyová M, Stúpalová S, Necas S. Sheep milk-borne epidemic of tick-borne encephalitis in Slovakia. Intervirology. 1975;5:57–61. 10.1159/000149880 1237478

[5] Kohl I, Kožuch O, Elecková E, Labuda M, Žaludko J. Family outbreak of alimentary tick-borne encephalitis in Slovakia associated with a natural focus of infection. Eur J Epidemiol. 1996;12:373–5. 10.1007/BF00145300 8891541

[6] Vereta LA, Skorobrekha VZ, Nikolaeva SP, Aleksandrov VI, Tolstonogova VI, Zakharycheva TA, et al. [The transmission of the tick-borne encephalitis virus via cow’s milk] [in Russian]. Med Parazitol (Mosk). 1991;3:54–6. 1770888

[7] Juceviciene A, Vapalahti O, Laiskonis A, Ceplikiene J, Leinikki P. Prevalence of tick-borne-encephalitis virus antibodies in Lithuania. J Clin Virol. 2002;25:23–7. 10.1016/S1386-6532(01)00215-3 12126718

[8] Kerbo N, Donchenko I, Kutsar K, Vasilenko V. Tickborne encephalitis outbreak in Estonia linked to raw goat milk, May-June 2005. Euro Surveill. 2005;10:E050623.2. 16783104 10.2807/esw.10.25.02730-en

[9] Holzmann H, Aberle SW, Stiasny K, Werner P, Mischak A, Zainer B, et al. Tick-borne encephalitis from eating goat cheese in a mountain region of Austria. Emerg Infect Dis. 2009;15:1671–3. 10.3201/eid1510.090743 19861072 PMC2866415

[10] Balogh Z, Ferenczi E, Szeles K, Stefanoff P, Gut W, Szomor KN, et al. Tick-borne encephalitis outbreak in Hungary due to consumption of raw goat milk. J Virol Methods. 2010;163:481–5. 10.1016/j.jviromet.2009.10.003 19836419

[11] Ličková M, Fumačová Havlíková S, Sláviková M, Klempa B. Alimentary infections by tick-borne encephalitis virus. Viruses. 2021;14:56. 10.3390/v14010056 35062261 PMC8779402

[12] Buczek AM, Buczek W, Buczek A, Wysokińska-Miszczuk J. Food-borne transmission of tick-borne encephalitis virus—spread, consequences, and prophylaxis. Int J Environ Res Public Health. 2022;19:1812. 10.3390/ijerph19031812 35162837 PMC8835261

[13] Shamseer L, Moher D, Clarke M, Ghersi D, Liberati A, Petticrew M, et al.; PRISMA-P Group. Preferred reporting items for systematic review and meta-analysis protocols (PRISMA-P) 2015: elaboration and explanation. BMJ. 2015;350(jan02 1):g7647. 10.1136/bmj.g7647 25555855

[14] Kerlik J, Avdičová M, Štefkovičová M, Tarkovská V, Pántiková Valachová M, Molčányi T, et al. Slovakia reports highest occurrence of alimentary tick-borne encephalitis in Europe: Analysis of tick-borne encephalitis outbreaks in Slovakia during 2007-2016. Travel Med Infect Dis. 2018;26:37–42. 10.1016/j.tmaid.2018.07.001 30012472

[15] Dorko E, Hockicko J, Rimárová K, Bušová A, Popaďák P, Popaďáková J, et al. Milk outbreaks of tick-borne encephalitis in Slovakia, 2012-2016. Cent Eur J Public Health. 2018;26(Suppl):S47–50. 10.21101/cejph.a5272 30817873

[16] Aendekerk RP, Schrivers AN, Koehler PJ. Tick-borne encephalitis complicated by a polio-like syndrome following a holiday in central Europe. Clin Neurol Neurosurg. 1996;98:262–4. 10.1016/0303-8467(96)00030-3 8884102

[17] Bartłomiej Borawski, Anna Nowicka-Ciełuszecka, Jadwiga Tarasiuk, Joanna Zajkowska, Zajkowska J; Monika Emilia Król. Outbreak of alimentary tick-borne encephalitis in Podlaskie voivodeship, Poland. Przegl Epidemiol. 2019;73:239–48. 31385681 10.32394/pe.73.01

[18] Matuszczyk I, Tarnowska H, Zabicka J, Gut W. [The outbreak of an epidemic of tick-borne encephalitis in Kielec province induced by milk ingestion] [in Polish]. Przegl Epidemiol. 1997;51:381–8. 9562785

[19] Caini S, Szomor K, Ferenczi E, Szekelyne Gaspar A, Csohan A, Krisztalovics K, et al. Tick-borne encephalitis transmitted by unpasteurised cow milk in western Hungary, September to October 2011. Euro Surveill. 2012;17:20128. 10.2807/ese.17.12.20128-en 22490310

[20] Camprubí D, Moreno-García E, Almuedo-Riera A, Martinez MJ, Navarro A, Martinez-Hernandez E, et al. First imported case of tick-borne encephalitis in Spain - was it alimentary? Travel Med Infect Dis. 2020;37:101701. 10.1016/j.tmaid.2020.101701 32339672

[21] Chitimia-Dobler L, Lindau A, Oehme R, Bestehorn-Willmann M, Antwerpen M, Drehmann M, et al. Tick-borne encephalitis vaccination protects from alimentary TBE infection: results from an alimentary outbreak. Microorganisms. 2021;9:889. 10.3390/microorganisms9050889 33919318 PMC8143337

[22] Brockmann SO, Oehme R, Buckenmaier T, Beer M, Jeffery-Smith A, Spannenkrebs M, et al. A cluster of two human cases of tick-borne encephalitis (TBE) transmitted by unpasteurised goat milk and cheese in Germany, May 2016. Euro Surveill. 2018;23:17–00336. 10.2807/1560-7917.ES.2018.23.15.17-00336 29667575 PMC6836198

[23] Markovinović L, Kosanović Ličina ML, Tešić V, Vojvodić D, Vladušić Lucić I, Kniewald T, et al. An outbreak of tick-borne encephalitis associated with raw goat milk and cheese consumption, Croatia, 2015. Infection. 2016;44:661–5. 10.1007/s15010-016-0917-8 27364148

[24] Ilic M, Barbic L, Bogdanic M, Tabain I, Savic V, Kosanovic Licina ML, et al. Tick-borne encephalitis outbreak following raw goat milk consumption in a new micro-location, Croatia, June 2019. Ticks Tick Borne Dis. 2020;11:101513. 10.1016/j.ttbdis.2020.101513 32993933

[25] Sixl W, Stünzner D, Withalm H, Köck M. Rare transmission mode of FSME (tick-borne encephalitis) by goat’s milk. Geogr Med Suppl. 1989;2:11–4. 2744469

[26] Hudopisk N, Korva M, Janet E, Simetinger M, Grgič-Vitek M, Gubenšek J, et al. Tick-borne encephalitis associated with consumption of raw goat milk, Slovenia, 2012. Emerg Infect Dis. 2013;19:806–8. 10.3201/eid1905.121442 23697658 PMC3647507

[27] Daniel M, Kríz B, Danielová V, Valter J, Kott I. Correlation between meteorological factors and tick-borne encephalitis incidence in the Czech Republic. Parasitol Res. 2008;103(Suppl 1):S97–107. 10.1007/s00436-008-1061-x 19030891

[28] Amicizia D, Domnich A, Panatto D, Lai PL, Cristina ML, Avio U, et al. Epidemiology of tick-borne encephalitis (TBE) in Europe and its prevention by available vaccines. Hum Vaccin Immunother. 2013;9:1163–71. 10.4161/hv.23802 23377671 PMC3899155

[29] Czupryna P, Moniuszko A, Pancewicz SA, Grygorczuk S, Kondrusik M, Zajkowska J. Tick-borne encephalitis in Poland in years 1993-2008—epidemiology and clinical presentation. A retrospective study of 687 patients. Eur J Neurol. 2011;18:673–9. 10.1111/j.1468-1331.2010.03278.x 21143706

[30] Beauté J, Spiteri G, Warns-Petit E, Zeller H. Tick-borne encephalitis in Europe, 2012 to 2016. Euro Surveill. 2018;23:1800201. 10.2807/1560-7917.ES.2018.23.45.1800201 30424829 PMC6234529

[31] Korenberg EI, Gorban LY, Kovalevskii YV, Frizen VI, Karavanov AS. Risk for human tick-borne encephalitis, borrelioses, and double infection in the pre-Ural region of Russia. Emerg Infect Dis. 2001;7:459–62. 10.3201/eid0703.017319 11384529 PMC2631802

[32] European Centre for Disease Prevention and Control. Brucellosis—annual epidemiological report for 2017 [cited 2019 Jun 19]. https://www.ecdc.europa.eu/en/publications-data/brucellosis-annual-epidemiological-report-2017 22114980

[33] Süss J. Epidemiology and ecology of TBE relevant to the production of effective vaccines. Vaccine. 2003;21(Suppl 1):S19–35. 10.1016/S0264-410X(02)00812-5 12628811

[34] Jaenson TG, Tälleklint L, Lundqvist L, Olsen B, Chirico J, Mejlon H. Geographical distribution, host associations, and vector roles of ticks (Acari: Ixodidae, Argasidae) in Sweden. J Med Entomol. 1994;31:240–56. 10.1093/jmedent/31.2.240 8189415 PMC7107449

[35] Golovljova I, Vene S, Sjölander KB, Vasilenko V, Plyusnin A, Lundkvist A. Characterization of tick-borne encephalitis virus from Estonia. J Med Virol. 2004;74:580–8. 10.1002/jmv.20224 15484275

[36] Haglund M, Vene S, Forsgren M, Günther G, Johansson B, Niedrig M, et al. Characterisation of human tick-borne encephalitis virus from Sweden. J Med Virol. 2003;71:610–21. 10.1002/jmv.10497 14556277

[37] Gonzalez G, Bournez L, Moraes RA, Marine D, Galon C, Vorimore F, et al. A One-Health approach to investigating an outbreak of alimentary tick-borne encephalitis in a non-endemic area in France (Ain, Eastern France): a longitudinal serological study in livestock, detection in ticks, and the first tick-borne encephalitis virus isolation and molecular characterisation. Front Microbiol. 2022;13:863725. 10.3389/fmicb.2022.863725 35479640 PMC9037541

[38] Bogovic P, Strle F. Tick-borne encephalitis: A review of epidemiology, clinical characteristics, and management. World J Clin Cases. 2015;3:430–41. 10.12998/wjcc.v3.i5.430 25984517 PMC4419106

[39] Kaiser R. The clinical and epidemiological profile of tick-borne encephalitis in southern Germany 1994-98: a prospective study of 656 patients. Brain. 1999;122:2067–78. 10.1093/brain/122.11.2067 10545392

