# Supplementary material for: Systematic Review and Meta-analysis of Foodborne Tick-Borne Encephalitis, Europe, 1980–2021
Source: Emerg Infect Dis. 2022 Oct;28(10):1945–54. doi: 10.3201/eid2810.220498 (PMC9514354; doi:10.3201/eid2810.220498)
Supplement: Appendix — Additional information on foodborne tick-borne encephalitis, Europe, 1980–2021. [file 22-0498-Techapp-s1.pdf]

Article DOI: https://doi.org/10.3201/eid2810.220498

Systematic Review and Metaanalysis of Foodborne Tickborne Encephalitis, Europe, 1980–2021

Appendix

Appendix Table. Laboratory-confirmed cases of foodborne tickborne encephalitis cases, Europe, 1980–2021*

Country (ref.)	No. cases	No. symptomatic cases	Blood serology	TBEV neutralization test	Serum rRT-PCR	HI test
Austria (*1*)	2	2	NA	NA	NA	NA
Austria (*2*)	6	4	Positive IgG and IgM for all 6	All 6 positive	NA	NA
Croatia (*3*)	7	7	Positive IgG and IgM for all 7	NA	NA	NA
Croatia (*4*)	5	0	NA	NA	NA	NA
Czech Republic (*5*)	2	0	NA	NA	NA	NA
Czech Republic (*5*)	1	0	NA	NA	NA	NA
Czech Republic (*5*)	28	0	NA	NA	NA	NA
Czech Republic (*5*)	5	0	NA	NA	NA	NA
Czech Republic (*5*)	6	0	NA	NA	NA	NA
Czech Republic (*5*)	2	0	NA	NA	NA	NA
Czech Republic (*5*)	8	0	NA	NA	NA	NA
Czech Republic (*5*)	8	0	NA	NA	NA	NA
Czech Republic (*5*)	4	0	NA	NA	NA	NA
Czech Republic (*6*)	1	1	Positive IgM	NA	NA	NA
Estonia (*7*)	1	0	Positive IgG and IgM	NA	NA	NA
Estonia (*8*)	27	0	NA†	NA	NA	NA
Germany (*9*)	14	14	Positive serology	NA	NA	NA
Germany (*10*)	2	2	Positive IgG and IgM for both	NA	NA	NA
Hungary (*11*)	25	25	Positive IgG and IgM for all 25	NA	NA	25/25
Hungary (*12*)	7	7	Positive IgM in blood or CSF in all 7	NA	NA	NA
Poland (*13*)	35	35	Positive IgG and IgM for all 35	NA	NA	NA
Poland (*14*)	4	4	2 Patients had elevated IgG and IgM; 1 had only elevated IgM; the fourth wasn't examined	NA	NA	NA
Russia (*15*)	5	5	Positive serology	NA	NA	NA
Slovakia (*16*)	169‡	0	NA	NA	NA	NA
Slovakia (*17*)	12	0	Positive IGM	NA	NA	NA
Slovakia (*17*)	3	0	Positive IGM	NA	NA	NA
Slovakia (*17*)	5	0	Positive IGM	NA	NA	NA
Slovakia (*17*)	11	0	NA	NA	NA	NA
Slovakia (*17*)	7	0	NA	NA	NA	NA
Slovakia (*17*)	3	0	NA	NA	NA	NA
Slovakia (*17*)	2	0	NA	NA	NA	NA
Slovakia (*17*)	2	0	Positive IGM	NA	NA	NA
Slovakia (*17*)	43	43	Positive IgG and IgM for all 43	NA	NA	NA
Slovakia (*17*)	5	0	NA	NA	NA	NA
Slovakia (*17*)	4	0	NA	NA	NA	NA
Slovakia (*17*)	2	0	NA	NA	NA	NA
Slovakia (*17*)	10	0	NA	NA	NA	NA
Slovakia (*18*)	7	7	Positive serology	NA	NA	NA
Slovenia (*19*)	3	3	Positive IgG and IgM for all 3	All 3 positive	All 3 negative	NA

*CSF, cerebrospinal fluid; HI, hemagglutination inhibition test; NA, not available; ref., reference number; rRT-PCR, real-time reverse transcription PCR; TBEV, tickborne encephalitis virus.
† The referenced text mentioned that all were laboratory confirmed.
‡109 of the 169 cases were mentioned in previous studies.

References

1. Sixl W, Stünzner D, Withalm H, Köck M. Rare transmission mode of FSME (tick-borne encephalitis) by goat's milk. Geogr Med Suppl. 1989;2:11–4. PubMed

2. Holzmann H, Aberle SW, Stiasny K, Werner P, Mischak A, Zainer B, et al. Tick-borne encephalitis from eating goat cheese in a mountain region of Austria. Emerg Infect Dis. 2009;15:1671–3. PubMed https://doi.org/10.3201/eid1510.090743

3. Markovinović L, Kosanović Ličina ML, Tešić V, Vojvodić D, Vladušić Lucić I, Kniewald T, et al. An outbreak of tick-borne encephalitis associated with raw goat milk and cheese consumption, Croatia, 2015. Infection. 2016;44:661–5. PubMed https://doi.org/10.1007/s15010-016-0917-8

4. Ilic M, Barbic L, Bogdanic M, Tabain I, Savic V, Kosanovic Licina ML, et al. Tick-borne encephalitis outbreak following raw goat milk consumption in a new micro-location, Croatia, June 2019. Ticks Tick Borne Dis. 2020;11:101513. PubMed https://doi.org/10.1016/j.ttbdis.2020.101513

5. Kríz B, Benes C, Daniel M. Alimentary transmission of tick-borne encephalitis in the Czech Republic (1997–2008). Epidemiol Mikrobiol Imunol. 2009;58:98–103. PubMed

6. Aendekerk RP, Schrivers AN, Koehler PJ. Tick-borne encephalitis complicated by a polio-like syndrome following a holiday in central Europe. Clin Neurol Neurosurg. 1996;98:262–4. PubMed https://doi.org/10.1016/0303-8467(96)00030-3

7. Camprubí D, Moreno-García E, Almuedo-Riera A, Martinez MJ, Navarro A, Martinez-Hernandez E, et al. First imported case of tick-borne encephalitis in Spain - was it alimentary? Travel Med Infect Dis. 2020;37:101701. PubMed https://doi.org/10.1016/j.tmaid.2020.101701

8. Kerbo N, Donchenko I, Kutsar K, Vasilenko V. Tickborne encephalitis outbreak in Estonia linked to raw goat milk, May–June 2005. Euro Surveill. 2005;10:E050623.2. PubMed https://doi.org/10.2807/esw.10.25.02730-en

9. Chitimia-Dobler L, Lindau A, Oehme R, Bestehorn-Willmann M, Antwerpen M, Drehmann M, et al. Tick-borne encephalitis vaccination protects from alimentary TBE infection: results from an alimentary outbreak. Microorganisms. 2021;9:889. PubMed https://doi.org/10.3390/microorganisms9050889

10. Brockmann SO, Oehme R, Buckenmaier T, Beer M, Jeffery-Smith A, Spannenkrebs M, et al. A cluster of two human cases of tick-borne encephalitis (TBE) transmitted by unpasteurised goat milk and cheese in Germany, May 2016. Euro Surveill. 2018;23:17-00336. PubMed https://doi.org/10.2807/1560-7917.ES.2018.23.15.17-00336

11. Balogh Z, Ferenczi E, Szeles K, Stefanoff P, Gut W, Szomor KN, et al. Tick-borne encephalitis outbreak in Hungary due to consumption of raw goat milk. J Virol Methods. 2010;163:481–5. PubMed https://doi.org/10.1016/j.jviromet.2009.10.003

12. Caini S, Szomor K, Ferenczi E, Szekelyne Gaspar A, Csohan A, Krisztalovics K, et al. Tick-borne encephalitis transmitted by unpasteurised cow milk in western Hungary, September to October 2011. Euro Surveill. 2012;17:20128. PubMed https://doi.org/10.2807/ese.17.12.20128-en

12. Matuszczyk I, Tarnowska H, Zabicka J, Gut W. The outbreak of an epidemic of tick-borne encephalitis in Kielec province induced by milk ingestion [in Polish]. Przegl Epidemiol. 1997;51:381–8. PubMed

14. Czupryna P, Moniuszko A, Pancewicz SA, Grygorczuk S, Kondrusik M, Zajkowska J. Tick-borne encephalitis in Poland in years 1993–2008—epidemiology and clinical presentation. A retrospective study of 687 patients. Eur J Neurol. 2011;18:673–9. PubMed https://doi.org/10.1111/j.1468-1331.2010.03278.x

15. Vereta LA, Skorobrekha VZ, Nikolaeva SP, Aleksandrov VI, Tolstonogova VI, Zakharycheva TA, et al. The transmission of the tick-borne encephalitis virus via cow's milk [in Russian.]. Med Parazitol (Mosk). 1991;3:54–6. PubMed

16. Kerlik J, Avdičová M, Štefkovičová M, Tarkovská V, Pántiková Valachová M, Molčányi T, et al. Slovakia reports highest occurrence of alimentary tick-borne encephalitis in Europe: analysis of tick-borne encephalitis outbreaks in Slovakia during 2007–2016. Travel Med Infect Dis. 2018;26:37–42. PubMed https://doi.org/10.1016/j.tmaid.2018.07.001

17. Dorko E, Hockicko J, Rimárová K, Bušová A, Popaďák P, Popaďáková J, et al. Milk outbreaks of tick-borne encephalitis in Slovakia, 2012-2016. Cent Eur J Public Health. 2018;26:S47–50. PubMed https://doi.org/10.21101/cejph.a5272

18. Kohl I, Kožuch O, Elecková E, Labuda M, Žaludko J. Family outbreak of alimentary tick-borne encephalitis in Slovakia associated with a natural focus of infection. Eur J Epidemiol. 1996;12:373–5. PubMed https://doi.org/10.1007/BF00145300

19. Hudopisk N, Korva M, Janet E, Simetinger M, Grgič-Vitek M, Gubenšek J, et al. Tick-borne encephalitis associated with consumption of raw goat milk, Slovenia, 2012. Emerg Infect Dis. 2013;19:806–8. PubMed https://doi.org/10.3201/eid1905.121442
